# Tuning the Slide-Roll Motion Mode of Carbon Nanotubes via Hydroxyl Groups

**DOI:** 10.1186/s11671-018-2554-x

**Published:** 2018-05-08

**Authors:** Rui Li, Shiwei Wang, Qing Peng

**Affiliations:** 1 School of Mechanical Engineering, University of Science and Technology Beijing, Beijing, 100083 China; 2 Nuclear Engineering and Radiological Sciences, University of Michigan, Ann Arbor, MI 48109 USA

**Keywords:** Carbon nanotubes, Hydroxyl groups, Motion control, Molecular dynamics simulations

## Abstract

Controlling the motion of carbon nanotubes is critical in manipulating nanodevices, including nanorobots. Herein, we investigate the motion behavior of SWCNT (10,10) on Si substrate utilizing molecular dynamics simulations. We show that hydroxyl groups have sensitive effect on the carbon nanotube’s motion mode. When the hydroxyl groups’ ratio on carbon nanotube and silicon substrate surfaces is larger than 10 and 20%, respectively, the motion of carbon nanotube transforms from sliding to rolling. When the hydroxyl groups’ ratio is smaller, the slide or roll mode can be controlled by the speed of carbon nanotube, which is ultimately determined by the competition between the interface potential energy and kinetic energy. The change of motion mode holds true for different carbon nanotubes with hydroxyl groups. The chirality has little effect on the motion behavior, as opposed to the diameter, attributed to the hydroxyl groups’ ratio. Our study suggests a new route to control the motion behavior of carbon nanotube via hydroxyl groups.

**Electronic supplementary material:**

The online version of this article (10.1186/s11671-018-2554-x) contains supplementary material, which is available to authorized users.

## Background

Controlling the motion behaviors of nanoelectromechanical systems (NEMS) and nanorobots is a complex and challenging issue due to surface and interface effects. The stimulation of natural, synthetic, physical, and other energy sources can control mechanical movement of nano- and micromotors [1]. For example, it is possible to drive nonpolar nanocars unidirectionally [2] and four-wheeled molecules directionally with the help of an external electric field [3] and thermally drive molecular nanocars directionally [4].

Carbon nanotubes play an important role in NEMS because of their excellent electrical, mechanical, and thermal properties. Nanodevices based on carbon nanotubes such as nanogears [5], nanomotors [6, 7], nanobearings [8, 9], and nanoscale electromechanical actuators [10] have been designed. However, tuning the motion of these nanodevices is still an open question. Research documents reported that thermal gradient was used to actuate the coaxial nanotubes [11]. Meanwhile, researchers studied the factors that might influence the motion behavior of carbon nanotubes, including the commensurate or incommensurate state between interfaces [12], the deformation of the carbon nanotubes [13–15], and the introduced groups such as hydrogens at the ends of a motor [16]. Among these factors, introducing functional groups on carbon nanotubes is relatively easy to achieve. Researchers have studied the motion and friction properties of surface-fluorinated carbon nanotubes [17], graphene oxide layers with different functional groups [18], and hydrogenated graphene [19, 20]. However, the effect of introduced hydroxyl groups on the motion behavior of carbon nanotubes has not been reported until now. This paper demonstrates that the introduction of hydroxyl groups can tune the rolling or sliding behavior of carbon nanotube. Our study may shed light on directionally controlled motion of sophisticated molecular mechanical systems based on carbon nanotubes, such as rack-and-pinion nanogear. Moreover, for other cylindrical nanomaterials, such as nanoscrolls that have great potential [21], the results also provide a possible way for the control of their motion.

## Methods

**Fig. 1:**
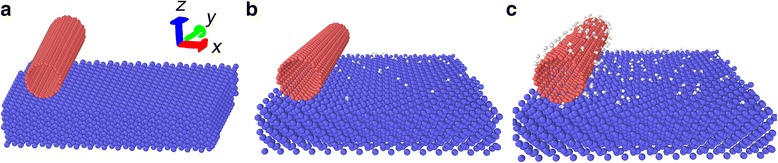
Simulation models. **a** Ideal. **b** Si substrate covered with hydroxyl groups. **c** Carbon nanotube and Si substrate are both covered with hydroxyl groups

The simulation models are composed of single-walled carbon nanotubes (10,10) (SWCNTs) and Si substrate. Three different structures are considered, as shown in Fig. 1. Model a is an ideal simulation model (Fig. 1a), which includes horizontally oriented carbon nanotube and Si substrate. Model b is composed of carbon nanotube and hydroxyl group-covered Si substrate (Fig. 1b). Model c is also composed of carbon nanotube and Si substrate, but both parts are covered with hydroxyl groups on the surfaces (Fig. 1c). The content of hydroxyl groups on the Si substrate refers to the ratio of the number of hydroxyl groups to the number of Si atoms on the surface of the Si substrate. The dimension of Si (0 0 1) substrate is 8.01 nm in the *x* direction and 7.98 nm in the *y* direction. The Si substrate consists of 5400 Si atoms.

The AIREBO potential [22] and TERSOFF potential [23] are applied to describe the interactions among C atoms within the carbon nanotube and those among Si atoms within the substrate, respectively. Since the O atoms are not considered in the AIREBO potential, an OPLS force field is employed to describe Si–O–H in Si substrate and C–O–H on carbon nanotube [24–27]. The hydrogen bond between interfaces in model c is calculated by the DREIDING force field [28]. The Van der Waals’ force between the carbon nanotube and Si substrate is described by Classic 12-6 Lennard-Jones (L-J) potential [29]. The parameters for C, H, and O can be found in literature [25], and the parameters for Si are in literature [28]. The motion of the carbon nanotube presented here is simulated by Large-Scale Atomic/Molecular Massively Parallel Simulator (LAMMPS) [30]. All simulations are performed in the canonical ensemble (NVT). The system temperature is 300 K. Comparing the results using Nosé-Hoover thermostat and the Langevin thermostat in model a, it shows that the Langevin thermostat nearly influences the motion of carbon nanotube and makes the system reach the equilibrium easier. Therefore, the Langevin thermostat is adopted in simulations. The damping coefficient of the Langevin thermostat, *t*_r_, which referred to the contribution from random forces in the Langevin equation, is set at 0.1 ps for all cases [31]. The bottom layer atoms of Si substrate are fixed to simulate Si wafer. The periodic boundary conditions are applied along the *x* and *y* directions. To conduct the same periodic boundary for carbon nanotube and Si substrate in the *y* direction, Si substrate is compressed 1.90% along the *y* direction, which is small; therefore, the influence on carbon nanotube’s motion can be ignored. The numerical integration of the equations of dynamic is performed by the Velocity-Verlet algorithm with a time step of 0.001 ps. The simulation process is as follows. First, the structure of the simulation system is optimized through energy minimization. Then, the relaxation is conducted for 100 ps to assure the system reaches equilibrium. Finally, a constant velocity or a constant force on the carbon nanotube along the *x* direction is set to make it move on the Si substrate. The constant velocity along the *x* direction is conducted by setting the lateral force of the center of the carbon nanotube zero.

## Results and Discussion

**Fig. 2:**
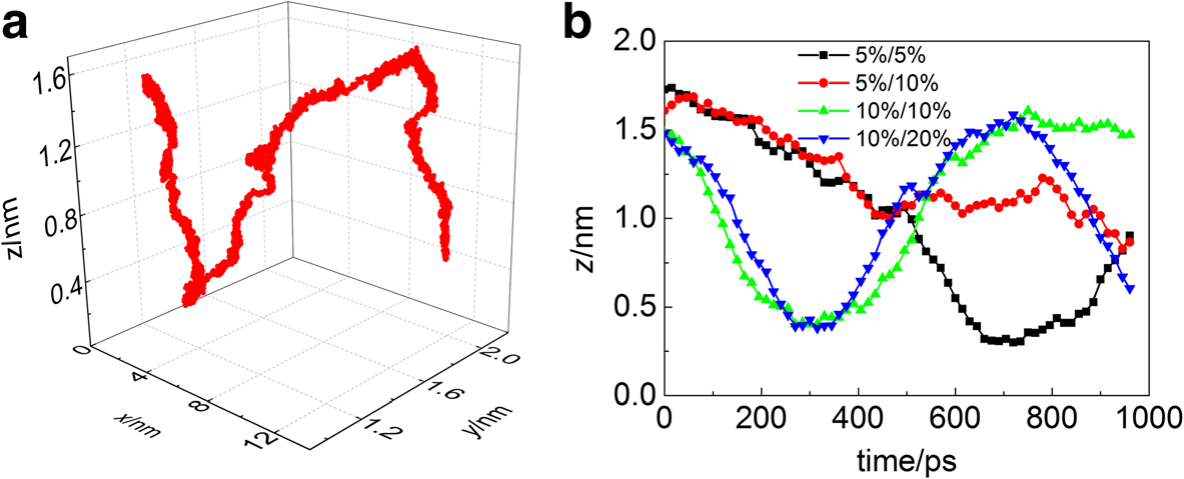
**a** The three-dimensional motion trajectory of a C atom on carbon nanotube. The hydroxyl groups’ ratio on carbon nanotube and Si substrate is 10 and 20%, respectively. **b** The coordinate of a C atom on carbon nanotube in the *z* direction as a function of time. The hydroxyl groups’ ratio on carbon nanotube and Si substrate is 5%/5%, 5%/10%, 10%/10%, and 10%/20%, respectively


We first set a constant translational velocity 10 m/s for carbon nanotube in the *x* direction. In both models a and b, the carbon nanotube slides on the substrate. However, rolling occurs in model c where the carbon nanotube and the Si substrate are both covered with hydroxyl groups. When the hydroxyl groups’ ratio of carbon nanotube and Si substrate are both 10%, the carbon nanotube rolls on the Si substrate, accompanied by slight sliding (Additional file 1: Movie S1). Moreover, if the hydroxyl groups’ ratio on the carbon nanotube and Si substrate are 10 and 20%, respectively, the carbon nanotube keeps rolling on the Si substrate during the simulation time (Additional file 2: Movie S2). Figure 2a shows the three-dimensional motion trajectory of a C atom on carbon nanotube when the hydroxyl groups’ ratio on carbon nanotube and Si substrate are 10 and 20%, respectively. The motion of the C atom represents the motion of the carbon nanotube because the carbon nanotube will not change its shape obviously. Carbon nanotube’s coordinate in the *z* direction moves up and down obviously, and the maximum of *z* displacement is about 1.3 nm, which is similar to the diameter of SWCNT (10,10) of 1.38 nm. The result indicates the motion of rolling. The carbon nanotube moves about 10.8 nm in the *x* direction. Because the constant velocity 10 m/s in the *x* direction is applied to the carbon nanotube, which makes the carbon nanotube move 9.5 nm in the *x* direction during the 950-ps motion process. Therefore, the extra moving distance in the *x* direction is 1.3 nm. The value is equal to the maximum of z displacement, which indicates rolling is dominant in the motion. Besides, the slight sliding in the *y* direction also occurs. The reason can be attributed to the disequilibrium force along the axial direction of the carbon nanotube due to the random distribution of hydroxyl groups, which makes the carbon nanotube slide along the *y* direction. The similar phenomena can be found in another research work [31]. When the hydroxyl groups’ ratio on the carbon nanotube and Si substrate changes to 5% and 5%, and 5% and 10%, the movement of carbon nanotube becomes different. Figure 2b shows the positon of a C atom in the *z* direction when the hydroxyl groups’ ratio on carbon nanotube and Si substrate is 5%/5%, 5%/10%, 10%/10%, and 10%/20%, respectively. In the cases that the hydroxyl groups’ ratio is 5%/5% and 5%/10%, sliding is the major movement, accompanied by slight rolling. In the case when hydroxyl groups’ ratio is 5%/5%, the carbon nanotube slides about 500 ps accompanied by slight rolling and then rolls about 500 ps. In the case when hydroxyl groups’ ratio is 5%/10%, the carbon nanotube slides about 500 ps with slight rolling and then keeps sliding.

**Additional file 1: Movies S1**. (AVI 4439 kb)



**Additional file 2: Movies S2**. (AVI 4929 kb)


**Fig. 3:**
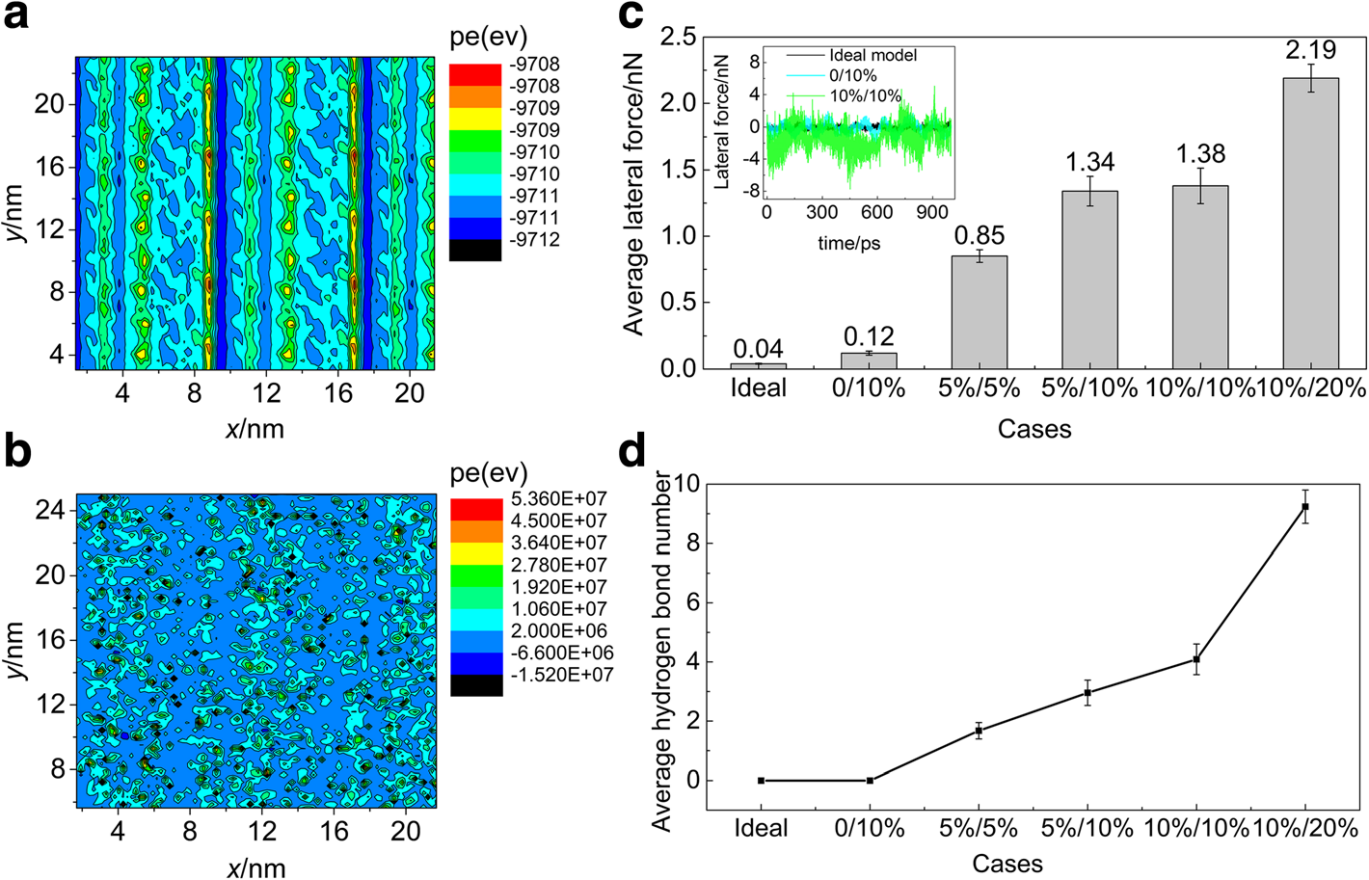
**a**, **b** The interfacial potential energy between carbon nanotube and Si substrate. **a** Ideal model. **b** The hydroxyl groups’ ratio on carbon nanotube and Si substrate is 10%/20%. **c** The average friction on carbon nanotube in the six cases. The inset shows friction of carbon nanotube with time in three cases in models a, b, and c. The hydroxyl groups’ ratio of carbon nanotube and Si substrate in models b and c is 0/10% and 10%/10%, respectively. **d** The average hydrogen bond numbers in the six cases in **c**

To establish the mechanism of the change of motion mode due to hydroxyl groups, we examine the interface potential energy under different conditions, as SWCNTs’ motion behavior is influenced by interface potential barrier [15]. The interfacial potential energies between carbon nanotube and Si substrate in models a and c are displayed in Fig. 3a, b, which is obtained by allowing the carbon nanotube slide over the substrate for 20.0 and 20.0 nm along the *x* and *y* directions, respectively, after relaxation. In model c, the case with the hydroxyl groups’ ratio of carbon nanotube and Si substrate 10%/20% is selected because the carbon nanotube keeps rolling under this condition. In the ideal model a, owing to the incommensurate state between the carbon nanotube and Si substrate, the distribution of the potential energy between interfaces is even. As a result, the carbon nanotube slides on the substrate. However, in model c, the interaction of hydroxyl groups between interfaces leads to an enormous change of interfacial potential energy. The peak of local potential barrier even reaches the order of 10^7^ eV. The random distribution of hydroxyl groups causes the uniform distribution of the high potential barrier. Therefore, the carbon nanotube cannot cross the potential barrier directly, resulting in rolling to reduce the interfacial potential barrier. Because the potential barrier covers the whole surface due to random distribution of hydroxyl groups, the carbon nanotube keeps rolling along the *x* direction. To the cases in which the ratio of hydroxyl groups of carbon nanotube and Si substrate is 5%/5%, 5%/10%, and 10%/10%, their potential barrier is relatively lower than the case in which the hydroxyl groups’ ratio is 10%/20%. The reason is that fewer hydroxyl groups on the interface result in weaker interaction. When the kinetic energy of carbon nanotube is higher than the barrier, it slides. Otherwise, the carbon nanotube begins to roll.

The introduction of hydroxyl groups between interfaces influences not only the motion of carbon nanotube but also the friction between interfaces. Figure 3c shows the average friction on carbon nanotube in six cases, where the hydroxyl groups’ ratio of the carbon nanotube and Si substrate is 0/0, 0/10%, 5%/5%, 5%/10%, 10%/10%, and 10%/20%, respectively. The results exhibit that the average friction increases significantly with the hydroxyl groups’ ratio. In models a and b, the average friction force is nearly zero. Since surface roughness increases due to the introduction of hydroxyl groups, average friction in model b is greater than that in ideal model a. The inset in Fig. 3c shows the fluctuation of lateral force in model b is larger than that in model a. In model c, because the carbon nanotube and Si substrate are both grafted hydroxyl groups, the fluctuation of lateral force and the mean friction are significantly greater than those in models a and b. When the hydroxyl groups’ ratio is 10%/20%, the mean friction increases to about 2.19 nN.

For more in-depth insights of the mechanism of the friction and motion behavior, we have studied the chemical bonds during motion. We observe that hydrogen bonds form between hydroxyl groups on interfaces. The corresponding average hydrogen bond numbers in these six cases are illustrated in Fig. 3d. The increment of the hydrogen bond number leads to higher potential barrier and friction with the increase of the hydroxyl groups’ ratio. This is in content that the hydrogen bond had great influence on friction [32].

**Fig. 4:**
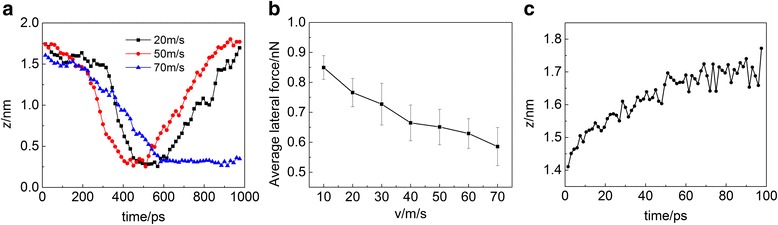
**a** The coordinate of a C atom on carbon nanotube in the *z* direction as a function of time when the carbon nanotube moves at speeds of 20, 50, and 70 m/s. **b** The mean friction forces’ curve with the velocities of carbon nanotube. **c** The coordinate of a C atom on the carbon nanotube in the *z* direction when the hydroxyl groups’ ratio on carbon nanotube and Si substrate is 5%/10%. The applied constant external force on the carbon nanotube is 0.000625 nN in the *x* direction

The motion behavior of carbon nanotube is influenced not only by the hydroxyl groups between interfaces but also by the velocity of carbon nanotubes, especially when an interfacial potential barrier is relatively low due to the small number of interfacial hydroxyl groups. With the carbon nanotube at speeds of 20, 50, 70 m/s, Fig. 4a shows the coordinate of a C atom in the *z* direction when the hydroxyl groups’ ratio of carbon nanotube and Si substrate is 5%/5%. At the speed of 20 m/s, rolling dominates in carbon nanotube’s motion. At the speed of 50 m/s, carbon nanotube moves 50 nm in the *x* direction and rolls for one round, which means sliding and rolling occur alternately. At the speed of 70 m/s, carbon nanotube mainly slides on the substrate accompanied by a slight rolling. The reason is similar to that the introduced hydroxyl groups between surfaces can tune the motion of carbon nanotube. Since the interface barrier is relatively low, when the kinetic energy of carbon nanotube is large, the carbon nanotube directly passes through it. However, when the kinetic energy is low, the carbon nanotube tends to roll to lower the interface barrier. Moreover, the curve of the mean friction force with velocity of carbon nanotube when the hydroxyl groups’ ratio is 5%/5% is shown in Fig. 4b. Friction decreases with velocity, which is consistent with other researchers’ experimental work [32].

A similar result can be obtained by applying a constant external force on carbon nanotube in the *x* direction. On the one hand, when the external force is large, the carbon nanotube only slides on the substrate. On the other hand, if the force is too small, the carbon nanotube cannot move. As a result, there is a roll-slide transition under a constant external force of 0.000625nN. Figure 4c shows the coordinate of a C atom on carbon nanotube in the *z* direction when the hydroxyl groups’ ratio of carbon nanotube and Si substrate is 5%/10%. The result shows that the coordinate of the C atom in the *z* direction increases obviously in the first stage, which indicates a rolling mode. Then, the coordinate in the *z* direction does not change much in the later stage, which means sliding mode dominates in the motion. The reason is that the kinetic energy of the carbon nanotube is small in the beginning, which is not able to overcome the interface barrier directly, resulting in rolling. With the increasing of the kinetic energy of the carbon nanotube, its motion behavior transforms from roll to slide.

**Fig. 5:**
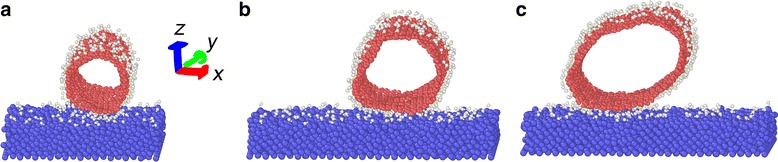
The structure of carbon nanotube on Si substrate. **a** SWCNT (15,15). **b** SWCNT (20,20). **c** SWCNT (25,25)

We further investigate the influence of chiral angle, diameter, and length of carbon nanotubes on their motion behaviors. First, we examine the chiral angle effect using five configurations, SWCNT (11,9), SWCNT (12,8), SWCNT (13,7), SWCNT (14,6), and SWCNT (15,0), which have varying angles but have almost the same diameters. The results show that their motion behavior is the same as that of SWCNT (10,10), indicating that the effect of chiral angle on the motion behavior of grafted hydroxyl carbon nanotubes can be neglected. Next, we select SWCNT (7,7), SWCNT (15,15), SWCNT (20,20), and SWCNT (25,25) to study the influence of diameter. The results of models a and b are similar to that of SWCNT (10,10). However, in model c, the results are different from that of SWCNT (10,10). When the motion mode of SWCNT (15,15), SWCNT (20,20), and SWCNT (25,25) changes to continuous rolling, the hydroxyl groups’ ratio is 10%/25%, 15%/30%, and 20%/30%, respectively. The larger the diameter, the higher the hydroxyl groups’ ratio when the motion mode changes. The reason can be attributed to the change of interface contact area. The interface structures show that SWCNT (15,15), SWCNT (20,20), and SWCNT (25,25) all have a platform on the bottom, as shown in Fig. 5, which causes the higher friction and the difficulty to roll. Higher ratio of hydroxyl groups offers stronger interface interaction and finally results in the occurrence of rolling. SWCNT (7,7) and SWCNT (10,10) both do not have a platform on the bottom, and then, the motion behavior of SWCNT (7,7) is almost the same as that of SWCNT (10,10). At last, we explore the length effect on motion by changing the length of SWCNT (10,10). Three lengths, 21.7, 54.3, and 81.4 nm, are explicitly scrutinized. We find that the motion behavior of SWCNT (10,10) with the length of 21.7 nm is consistent with the initial model c. However, in cases with lengths of 54.3 and 81.4 nm, they exhibit slight bending deformation during the rolling process due to large aspect ratio of length to diameter.

## Conclusions

In summary, we reveal that the introduction of hydroxyl groups between the interfaces leads to the formation of hydrogen bonds, which increases the interface barrier and friction. The motion mode (slide or roll) of carbon nanotube on Si substrate can be tuned by the introduced hydroxyl groups’ ratio on the interfaces and the speed of carbon nanotube. When the hydroxyl groups’ ratio on carbon nanotube and Si substrate are small (< 10%/20%), the motion of carbon nanotube depends on interface potential barrier and kinetic energy. If the kinetic energy of carbon nanotube is high, the carbon nanotube slides on the substrate. Otherwise the carbon nanotube tends to roll to lower the barrier. When the hydroxyl groups’ ratio on carbon nanotube and Si substrate is higher than 10%/20%, in which the interfacial potential energy barrier is very high, the carbon nanotube keeps rolling. The tuning of the motion mode is feasible to CNTs with different chiral angles, lengths, and diameters by adjusting the hydroxyl groups’ ratio. The effect of the hydroxyl group on the motion mode of the carbon nanotube could be used to control the motion of CNT, and programmable nanodevices could be fabricated.

## Abbreviations

LAMMPS: Large-Scale Atomic/Molecular Massively Parallel Simulator
NEMs: Nanoelectromechanical systems
SWCNTs: Single-walled carbon nanotubes

## References

[1] Guix M, Mayorga-Martinez CC, Merkoci A (2014). Nano/micromotors in (bio)chemical science applications. Chem Rev.

[2] Akimov AV, Kolomeisky AB (2012). Unidirectional rolling motion of nanocars induced by electric field. J Phys Chem C.

[3] Kudernac T, Ruangsupapichat N, Parschau M, Macia B, Katsonis N, Harutyunyan SR, Ernst KH, Feringa BL (2011). Electrically driven directional motion of a four-wheeled molecule on a metal surface. Nature.

[4] Shirai Y, Osgood AJ, Zhao YM, Kelly KF, Tour JM (2005). Directional control in thermally driven single-molecule nanocars. Nano Lett.

[5] Han J, Globus A, Jaffe R, Deardorff G (1997). Molecular dynamics simulations of carbon nanotube-based gears. Nanotechnology.

[6] Lohrasebi A, Raffii-Tabar H (2008). Computational modeling of an ion-driven nanomotor. J Mol Graph Model.

[7] Takagi Y, Uda T, Ohno T (2008). Carbon nanotube motors driven by carbon nanotube. J Chem Phys Physics.

[8] Isobe H, Hitosugi S, Yamasaki T, Iizuka R (2013). Molecular bearings of finite carbon nanotubes and fullerenes in ensemble rolling motion. Chem Sci.

[9] Zhang SL, Liu WK, Ruoff RS (2004). Atomistic simulations of double-walled carbon nanotubes (DWCNTs) as rotational bearings. Nano Lett.

[10] Fennimore AM, Yuzvinsky TD, Han WQ, Fuhrer MS, Cumings J, Zettl A (2003). Rotational actuators based on carbon nanotubes. Nature.

[11] Barreiro A, Rurali R, Hernandez ER, Moser J, Pichler T, Forro L, Bachtold A (2008). Subnanometer motion of cargoes driven by thermal gradients along carbon nanotubes. Science.

[12] Falvo MR, Steele J, Taylor RM, Superfine R (2000). Gearlike rolling motion mediated by commensurate contact: carbon nanotubes on HOPG. Phys Rev B.

[13] Buldum A, Lu JP (1999). Atomic scale sliding and rolling of carbon nanotubes. Phys Rev Lett.

[14] Schall JD, Brenner DW (2000). Molecular dynamics simulations of carbon nanotube rolling and sliding on graphite. Mol Simul.

[15] Chauveau V, Mazuyer D, Dassenoy F, Cayer-Barrioz J (2012). In situ film-forming and friction-reduction mechanisms for carbon-nanotube dispersions in lubrication. Tribol Lett.

[16] Gao Z, Cai H, Shi J, Liu L, Chen Z, Wang Y (2017). Effect of hydrogenation and curvature of rotor on the rotation transmission of a curved nanobearing. Comput Mater Sci.

[17] Vander Wal R, Miyoshi K, Street K, Tomasek A, Peng H, Liu Y, Margrave J, Khabashesku V (2005). Friction properties of surface-fluorinated carbon nanotubes. Wear.

[18] Wang L-F, Ma T-B, Hu Y-Z, Wang H (2012). Atomic-scale friction in graphene oxide: an interfacial interaction perspective from first-principles calculations. Phys Rev B.

[19] Dong YL, Wu XW, Martini A (2013). Atomic roughness enhanced friction on hydrogenated graphene. Nanotechnology.

[20] Wang JJ, Li JM, Fang LL, Sun Q, Jia Y (2014). Charge distribution view: large difference in friction performance between graphene and hydrogenated graphene systems. Tribol Lett.

[21] Cui X, Kong Z, Gao E, Huang D, Hao Y, Shen H, Di CA, Xu Z, Zheng J, Zhu DB (2018). Rolling up transition metal dichalcogenide nanoscrolls via one drop of ethanol. Nat Commun.

[22] Brenner DW, Shenderova OA, Harrison JA, Stuart SJ, Ni B, Sinnott SB (2002). A second-generation reactive empirical bond order (REBO) potential energy expression for hydrocarbons. J Phys Condes Matter.

[23] Tersoff J (1988). New empirical approach for the structure and energy of covalent systems. J Phys Rev B.

[24] Argyris D, Tummala NR, Striolo A, Cole DR (2008). Molecular structure and dynamics in thin water films at the silica and graphite surfaces. J Phys Chem C.

[25] Damm W, Frontera A, Tirado-Rives J, Jorgensen WL (1997). OPLS all-atom force field for carbohydrates. J Comput Chem.

[26] Hughes ZE, Shearer CJ, Shapter J, Gale JD (2012). Simulation of water transport through functionalized single-walled carbon nanotubes (SWCNTs). J Phys Chem C.

[27] Nishiyama K, Watanabe T, Hoshino T, Ohdomari I (2005). Analysis of interactions between green fluorescent protein and silicon substrates using molecular dynamics simulations. Jpn J Appl Phys.

[28] Mayo SL, Olafson BD, Goddard WA (1990). DREIDING: a generic force field for molecular simulations. J Phys Chem.

[29] Ruoff RS, Hickman AP (1993). Van der Waals binding of fullerenes to a graphite plane. J Phys Chem.

[30] Plimpton S (1995). Fast parallel algorithms for short-range molecular dynamics. J Comput Phys.

[31] Li R, Sun D, Zhang B (2014). Motion and energy dissipation of single-walled carbon nanotube on graphite by molecular dynamics simulation. Mater Res Express.

[32] Chen J, Ratera I, Park JY, Salmeron M (2006). Velocity dependence of friction and hydrogen bonding effects. Phys Rev Lett.

